# Diagnostic Value of the Ocular Syndrome Due to Hypertension in Wounds of the Skull

**Published:** 1919-01

**Authors:** 


**Diagnostic Value of the Ocular Syndrome Due to Hypertension in Wounds of the Skull.** By Dr. F. TERRIEN. *Paris Médical*, October 12, 1918.

In addition to the visual troubles due to lesions in the visual centers, a series of troubles solely due to intracranial hypertension may be observed in wounds of the skull, chiefly after trepanation.

These troubles are usually as transient as their cause. The accompanying general troubles may be of an insignificant nature and the vision remains excellent.

Ocular syndrome, due to hypertension, is characterized by three symptoms: transient diplopia, narrowing of the field of vision, and papillary stasis, which the author observed in a number of cases and which he summarizes as follows:

*Transient diplopia* is caused by a compression of the external motor oculi nerves, probably taking its origin in a transient cerebral hypertension, as is the case in alcoholism.

*The narrowing of the field of vision* is never sufficiently pronounced to inconvenience the patient.

*The papillary stasis* never reaches the degree of intensity of the stasis of cerebral tumors. Its manifestation is a series of subjective troubles characterized by transient obnubilations of the vision.

If proper measures are taken in time, no decrease in visual acuity need be feared, for the above described symptoms will disappear with the removal of hypertension, i. e., by means of lumbar punctures. The research of the ocular syndrome is, however, important from two standpoints: it permits the avoidance of permanent visual troubles, and facilitates the discovery of lesions which might, in some cases, be overlooked.

